# CFM-ID 3.0: Significantly Improved ESI-MS/MS Prediction and Compound Identification

**DOI:** 10.3390/metabo9040072

**Published:** 2019-04-13

**Authors:** Yannick Djoumbou-Feunang, Allison Pon, Naama Karu, Jiamin Zheng, Carin Li, David Arndt, Maheswor Gautam, Felicity Allen, David S. Wishart

**Affiliations:** 1 Department of Biological Sciences, University of Alberta, Edmonton, AB T6G 2E9, Canada; djoumbou@ualberta.ca (Y.D.-F.); n.karu@lacdr.leidenuniv.nl (N.K.); jiamin3@ualberta.ca (J.Z.); cbli@ualberta.ca (C.L.); darndt@ualberta.ca (D.A.); maheswor@ualberta.ca (M.G.); 2 OMx Personal Health Analytics, Edmonton, AB T5J 1B9, Canada; allisonpon@gmail.com; 3 Wellcome Sanger Institute, Wellcome Trust Genome Campus, Hinxton CB10 1SA, UK; felicity.allen@sanger.ac.uk; 4 Department of Computing Science, University of Alberta, Edmonton, AB T6G 2E8, Canada

**Keywords:** mass spectrometry, liquid chromatography, MS spectral prediction, metabolite identification, structure-based chemical classification, rule-based fragmentation, combinatorial fragmentation

## Abstract

Metabolite identification for untargeted metabolomics is often hampered by the lack of experimentally collected reference spectra from tandem mass spectrometry (MS/MS). To circumvent this problem, Competitive Fragmentation Modeling-ID (CFM-ID) was developed to accurately predict electrospray ionization-MS/MS (ESI-MS/MS) spectra from chemical structures and to aid in compound identification via MS/MS spectral matching. While earlier versions of CFM-ID performed very well, CFM-ID’s performance for predicting the MS/MS spectra of certain classes of compounds, including many lipids, was quite poor. Furthermore, CFM-ID’s compound identification capabilities were limited because it did not use experimentally available MS/MS spectra nor did it exploit metadata in its spectral matching algorithm. Here, we describe significant improvements to CFM-ID’s performance and speed. These include (1) the implementation of a rule-based fragmentation approach for lipid MS/MS spectral prediction, which greatly improves the speed and accuracy of CFM-ID; (2) the inclusion of experimental MS/MS spectra and other metadata to enhance CFM-ID’s compound identification abilities; (3) the development of new scoring functions that improves CFM-ID’s accuracy by 21.1%; and (4) the implementation of a chemical classification algorithm that correctly classifies unknown chemicals (based on their MS/MS spectra) in >80% of the cases. This improved version called CFM-ID 3.0 is freely available as a web server. Its source code is also accessible online.

## 1. Introduction

Liquid chromatography (LC) coupled to mass spectrometry (MS) or tandem mass spectrometry (MS/MS) has become one of the leading techniques for compound identification in organic chemistry, natural product chemistry, and metabolomics [1,2]. In the field of metabolomics, LC-MS/MS is widely used to identify and quantify individual chemicals in complex biological or environmental mixtures. For untargeted MS-based metabolomics, high performance or ultrahigh performance liquid chromatography (HPLC or UHPLC) is first performed to separate compounds in the sample and then electrospray ionization (ESI) mass spectrometry (MS and MS/MS) is used to collect the mass spectra of each chromatographic peak. In order to identify individual compounds, the resulting MS/MS spectra, along with the chromatographic retention time and parent ion masses of the compound of interest, are then (ideally) compared to the MS/MS spectra and retention time of authentic standards to confirm the compound’s identity.

Because of the limited availability of many authentic chemical standards in most metabolomics labs, putative metabolite identification is more commonly performed [3]. Putative identification (MSI level 2) is achieved by comparing the MS/MS spectra to experimentally collected reference spectra found in various MS/MS spectral databases. Key to the success of this putative identification process is the availability of a large, comprehensive database containing experimentally collected MS/MS spectra of pure compounds that covers a large portion of “chemical space”. Unfortunately, publicly available databases of experimental MS/MS spectra currently cover a total of only ~20,000 unique compounds [4]. Consequently, as reported in many large-scale metabolomic studies [5,6], the percentage of MS spectral features that can be confidently assigned to known compounds is often less than 2%. As a result, the compound identification step continues to be the central bottleneck in almost all untargeted MS-based metabolomic studies.

Given the cost of synthesizing or acquiring the 100,000’s of chemicals needed to create the required experimental MS/MS spectral libraries, a growing number of scientists are turning to in silico metabolomic methods to facilitate compound identification. Over the last decade, a number of computational MS approaches have been developed for this purpose. Some of the more popular software tools use MS/MS fragmentation trees and spectral fingerprints (e.g., CSI:FingerID [7]) of an observed ESI-MS/MS spectrum to rank the likelihood that a given chemical structure could produce such a spectrum. Other tools arrange substructures of a candidate molecule into a format that best explains the fragmentation pattern observed in a given experimental MS^n^ spectral tree (MAGMA [8]). Still others, such as MetFrag [9] use in silico fragmentation of candidate molecules, based on a given mass spectrum and mass of a precursor molecule to identify its structure. Competitive Fragmentation Modeling-ID (CFM-ID) [10,11,12] use in silico fragmentation techniques to predict ESI-MS/MS (for LC-MS) or EI-MS (for GC-MS) spectra for a given structure. By matching the observed MS/MS spectrum to a library of predicted MS/MS spectra, it is possible to identify or rank which compound is being observed. It is widely believed that increasing the number (and accuracy) of in silico-predicted spectra should increase the likelihood of successfully identifying compounds from newly acquired MS/MS spectra [13].

The two main in silico MS fragmentation techniques are rule-based approaches and combinatorial approaches. Rule-based “fragmenters” use hand-made rules based on experimentally observed fragmentation patterns that are specific to one or more structural features or chemical classes. These rules are typically extracted from analyzing the scientific literature or, preferably, learned from in-house experimental data. Mass Frontier™ (ThermoFisher, CA; HighChem, Bratislava, Slovakia) is an example of a software tool that uses hand-made fragmentation rules. Once the rules are implemented, this approach can be very fast, consistent, and accurate. However, a major disadvantage to this approach is that the design of fragmentation rules requires considerable expert curation. Furthermore, these rules cannot be applied to novel classes of molecules. For these reasons, much more emphasis has recently been put toward the implementation of computational combinatorial fragmentation approaches. Combinatorial fragmentation approaches iteratively cleave chemical bonds within a molecule in a combinatorial fashion, and use (or learn) penalty scores that favor the cleavage events that are most likely to occur at each step. Examples of tools that implement combinatorial fragmentation include CFM-ID [10,11,12], MetFrag [9], and FiD [14].

CFM-ID is a publicly available software tool and web server that can be used for MS/MS spectral prediction, MS/MS spectrum peak assignment, as well as MS-based compound identification. It implements a technique known as Competitive Fragmentation Modeling (CFM). CFM is a probabilistic generative modeling method that uses a customized cost function to take into account the structural composition of a molecule to predict its electrospray ESI-MS/MS spectrum. The original version of CFM-ID was used to generate a reference MS/MS spectral library of over 51,000 known compounds from the HMDB [15] and KEGG [16] databases at 3 different collision energies (10, 20, and 40 eV). This spectral library was used by CFM-ID (version 1.0 and version 2.0) to suggest candidate molecules that match input experimental MS/MS spectra. In 2015, the original version of CFM-ID was shown to outperform FingerID and an earlier version of MetFrag in various identification tasks from ESI-MS/MS spectra [11]. However, subsequent tests and studies that further assessed the performance of CFM-ID have shown that a number of improvements could be made to the program and its spectral database [11,12].

For instance, one well-known limitation of CFM-ID is its very slow and relatively poor performance for predicting MS/MS spectra of lipids and other large “segmented” or modular metabolites. This is primarily due to the length of the fatty acids or attached head-group segments, leading to a combinatorial explosion of the possible fragments at each step of the in silico fragmentation process. As demonstrated by Kind et al. [17], who developed LipidBlast, and Tsugawa et al. [18], who studied sphingolipid fragmentation, the use of structure-based fragmentation rules appears to be much better at handling lipids and other large segmented or modular molecules (such as carbohydrates) than combinatorial fragmentation. However, it is important to note that LipidBlast also has some limitations. For instance, it does not provide a well-defined set of fragmentation rules or algorithms that can be incorporated into other computational MS spectral prediction tools. Furthermore, while LipidBlast does provide *m*/*z* values and heuristically modeled static relative abundances for fragment ions, the annotation of fragment ion peaks is limited to formulas and does not include actual structures. Moreover, LipidBlast predict consensus mass spectra, and does not distinguish between different collision energies. These are the kinds of output that are typically found with most in silico fragmenters, and these shortcomings have been addressed in this update to CFM-ID.

In addition to the incorporation of compound-specific fragmentation rules, it has also been shown that significant improvements in MS-based compound identification can be achieved by including metadata or other forms of external data in the spectral matching and scoring functions [9]. In particular, the inclusion of citation frequency (the number of times a given compound is mentioned in the literature), along with the incorporation of experimentally collected MS/MS spectra in the reference spectral database can often improve compound identification performance by a factor of 2 or more [19]. When taking into account the chemical similarity or the distribution of structural features or chemical classes (via ClassyFire [20]) among candidates, it is often possible to improve the performance even further [7]. Based on these and other developments in the field of in silico metabolomics and in silico mass spectrometry, we have implemented a number of modifications to CFM-ID. These modifications have helped in a number of important ways, including (1) achieving faster and more accurate prediction of MS/MS-spectra for 21 classes of lipids, (2) enabling the expansion of CFM-ID’s reference spectral library to include both experimental and predicted MS/MS spectra, (3) enhancing CFM-ID’s ability to incorporate metadata and chemical similarity, (4) improving CFM-ID’s compound identification rates, and (5) enhancing CFM-ID’s ability to predict the structural class of compounds for query spectra that could not be matched in CFM-ID’s spectral database. The improved version of CFM-ID is called CFM-ID 3.0. It is freely available as a web server at http://cfmid3.wishartlab.com. Its source code is accessible at https://sourceforge.net/p/cfm-id/wiki/Home (combinatorial fragmentation tool) and https://bitbucket.org/wishartlab/msrb-fragmenter/ (rule-based fragmentation tool).

## 2. Results

### 2.1. Encoding Lipid Fragmentation Rules

**Figure 1:**
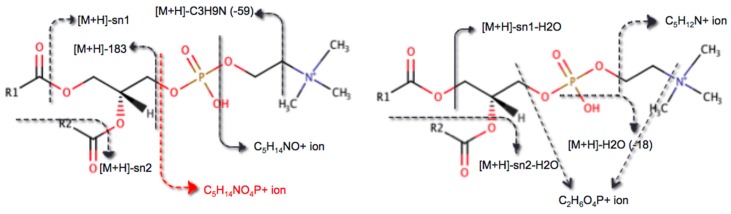
Fragmentation patterns of phosphatidylcholines obtained from their [M+H]^+^ precursor ions. Among all resulting fragments, only the precursor ion is observed at each of the three energy levels. The ion fragment C_5_H_14_NO_4_P+ (red arrow) corresponding to phosphocholine is observed at 20 and 40 eV, and the remaining fragments were observed only at 40 eV.

Our manual analysis of experimentally acquired lipid MS/MS spectra provided a basis for the generation of 344 unique fragmentation rules covering 21 lipid classes and 7 adducts, for a total of 50 combinations of lipid classes and adduct types. Each rule describes a chemical reaction that fragments the precursor molecule to generate a specific fragment. The structure and mass-to-charge ratio of the fragment can be easily computed based on the encoded pattern. For each lipid class, an ESI-MS/MS spectrum can be simulated by CFM-ID 3.0 at collision energies of 10, 20, and 40 eV. In general, almost all ESI-MS/MS spectra of lipids show similar fragmentation patterns with characteristic losses of the polar head group, and the acyl or alkyl chains, with relatively little fragmentation within the acyl or alkyl chains. For example, in choline-containing glycerophospholipids, the most commonly observed fragments include phosphocholine (C_5_H_14_NO_4_P+ ion; neutral mass = 184.0733 Da) and the cyclic 1,2-cyclic phosphate diester (C_2_H_6_O_4_P+ ion; neutral mass = 125.0003 Da). Figure 1 illustrates consensus fragmentation patterns for phosphatidylcholines from their [M+H]^+^ precursor ions. The number of rules for each lipid class and the number of covered adduct types per lipid class are shown in Table 1. These fragmentation rules are also available at https://bitbucket.org/wishartlab/msrb-fragmenter/.

### 2.2. The New CFM-ID 3.0 Spectral Library

The original CFM-ID 2.0 spectral library contained 102,153 unique computationally generated ESI-MS/MS spectra (from 51,635 compounds). Because of improvements in the spectral prediction performance, additions of new compounds, and the decision to add experimental spectra, the new CFM-ID 3.0 spectral library has been expanded by a factor of 2.6 over the original CFM-ID 2.0 spectral library (as of February 2019). In particular, the new library now contains a total of 167,547 computationally generated ESI-MS/MS spectra (generated via CFM-ID 3.0) from 108,972 compounds in the HMDB [15]; 22,914 computationally generated ESI-MS/MS spectra from 11,685 compounds in KEGG [16]; and 83,049 experimentally collected ESI-MS/MS spectra from 21,904 compounds. The compounds in CFM-ID 3.0’s experimental MS/MS spectral library are structurally and functionally diverse, and originate from various databases/libraries including HMDB (human metabolites) [15], DrugBank (drugs and drug metabolite) [21], KEGG (metabolites and drugs) [16], PhytoHub (dietary phytochemicals and their metabolites) [22], GNPS (natural products) [23], and MoNA [24]. In addition, 568 spectra from the CASMI 2014 [25] and CASMI 2016 [19] challenges were imported into the database. Moreover, 3953 spectra that were experimentally acquired at the Metabolomics Innovation Center (TMIC, Edmonton, AB, Canada) were also added. Each of the 229,084 compounds in the new spectral library was assigned a citation score (described below) that is used as metadata in compound identification tasks. Each compound in the spectral library has two or more citations. A summary of the library’s statistics is displayed in Table 2.

In our effort to improve the identification rates, a full chemical classification was computed for all 229,084 unique compounds using the computational chemical classifier called ClassyFire [20]. An average of ~25 chemical categories were assigned per compound. The chemical classification was used to adjust CFM-ID’s original scoring system, to take into account the chemical composition and chemical similarity among candidate molecules. This compound classification process also served as a basis to predict the chemical class of any new compound corresponding to the query spectrum in identification tasks.

### 2.3. Performance Testing

#### 2.3.1. Lipid ESI-MS/MS Spectral Prediction

Two tests were performed to assess CFM-ID 3.0’s lipid spectral prediction performance. One was for speed while the other was for accuracy. In terms of speed, CFM-ID 3.0 averaged 0.395 ± 0.03 s of computation time to predict each of the 120 lipid ESI-MS/MS spectra in the test set, while CFM-ID 2.0 averaged 68.58 ± 0.21 s for the same task. This represents a speed-up of 173.6X. Clearly, the rule-based approach for lipid analysis used in CFM-ID 3.0 is significantly faster than the combinatorial approach used in CFM-ID 2.0. For most other kinds of molecules, the average processing time for CFM-ID (3.0 and 2.0) is about 23.75 ± 0.2 s. Clearly, the computational slow-down for lipid spectral calculation (due to the many potential fragmentation combinations) is quite significant, which largely motivated us to develop a faster rule-based approach.

**Figure 2:**
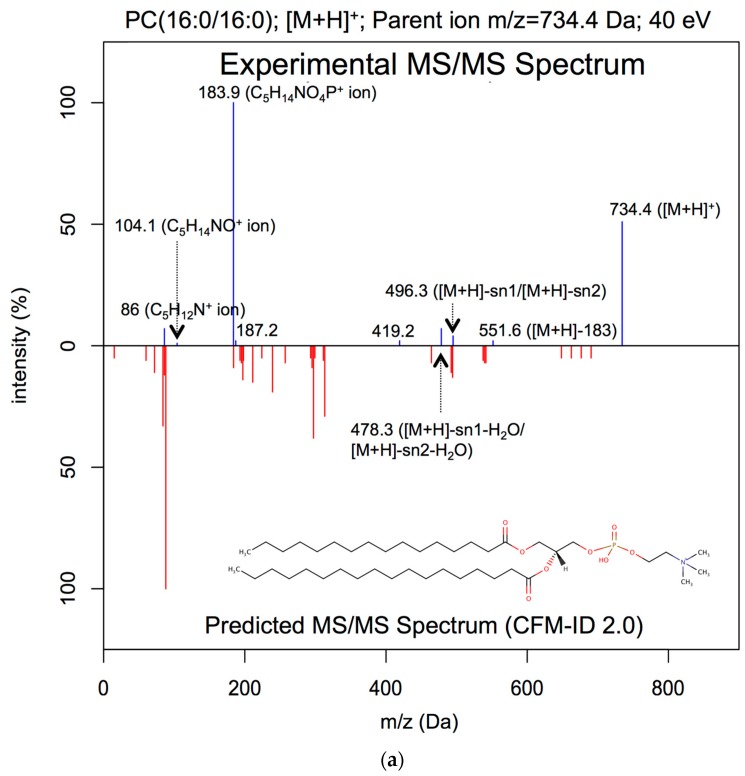

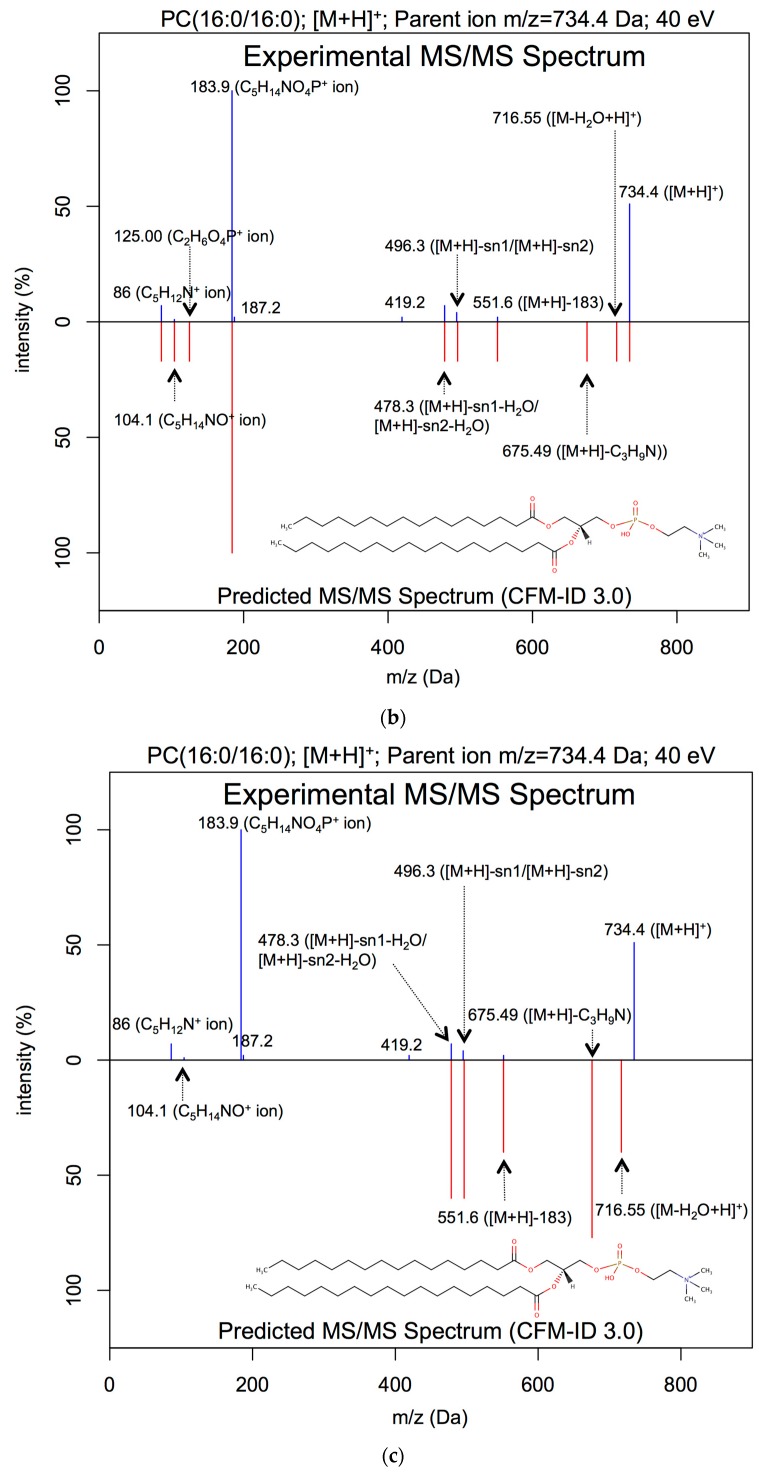
Head-to-tail plot of experimental and predicted electrospray ionization-tandem mass spectroscopy (ESI-MS/MS) spectra of PC(16:0/16:0). (**a**) Head-to-tail plot showing an experimental ESI-MS/MS spectrum of dipalmitoyl phosphatidylcholine (PC(16:0/16:0)) measured at 40 eV, and the matching ESI-MS/MS spectrum predicted by CFM-ID 2.0. The computed spectral similarity score is 0.07. (**b**) Head-to-tail plot showing an experimental of ESI-MS/MS spectrum of dipalmitoyl phosphatidylcholine measured in positive ion mode ([M+H]^+^) at 40 eV, and the matching ESI-MS/MS spectrum predicted by CFM-ID 3.0. The computed spectral similarity score is 0.88. (**c**) Head-to-tail plot showing an experimental of ESI-MS/MS spectrum of dipalmitoyl phosphatidylcholine measured in positive ion mode ([M+H]^+^) at 40 eV, and the matching ESI-MS/MS spectrum predicted by LipidBlast. The computed spectral similarity score is 0.13.

In terms of spectral prediction performance, the average spectral similarity score (measured using the dot product) between the experimental lipid ESI-MS/MS spectra (collected on a QTOF at multiple collision energies) and the CFM-ID 3.0-predicted ESI-MS/MS spectra was 0.85 ± 0.2. On the other hand, the average spectral similarity score between the CFM-ID 2.0-predicted ESI-MS/MS spectra and the experimental ESI-MS/MS spectra was 0.09 ± 0.1. This suggests that the accuracy of CFM-ID 3.0 for lipid spectral prediction is 11X better than that of CFM-ID 2.0, which is highly significant. It is worth mentioning that CFM-ID predicts ESI-MS/MS spectra at three different collision energies while other programs, such as LipidBlast, generate a consensus MS/MS spectrum that essentially merges the MS/MS spectra over all collision energies. Therefore, during our comparative analysis, only one LipidBlast-generated consensus ESI-MS/MS spectrum was used for each unique compound and compared against the experimental spectrum, independent of the energy level. The average spectral similarity score between the LipidBlast-predicted ESI-MS/MS spectra and the experimental ESI-MS/MS spectra was 0.34 ± 0.4. Figure 2 shows head-to-tail plots comparing the experimental ESI-MS/MS spectrum of dipalmitoyl phosphatidylcholine (PC(16:0/16:0)) collected at a 40 eV collision energy with the corresponding in silico spectra predicted with CFM-ID 2.0 [11] (Figure 2a), CFM-ID 3.0 (Figure 2b), and LipidBlast [17] (Figure 2c), respectively. The experimental spectrum was measured in positive ion mode ([M+H]^+^), with a collision energy of 40 eV.

**Figure 3:**
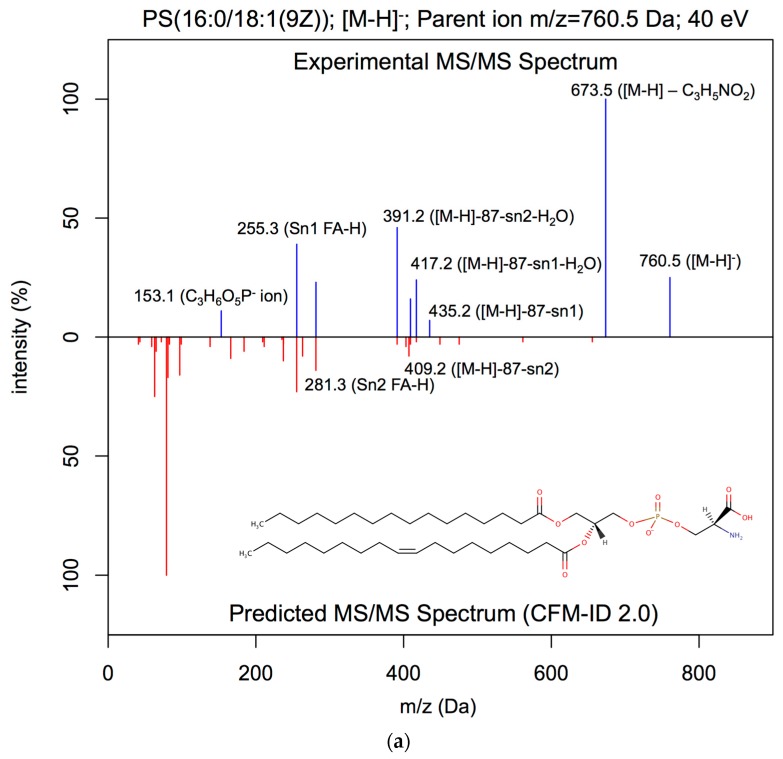

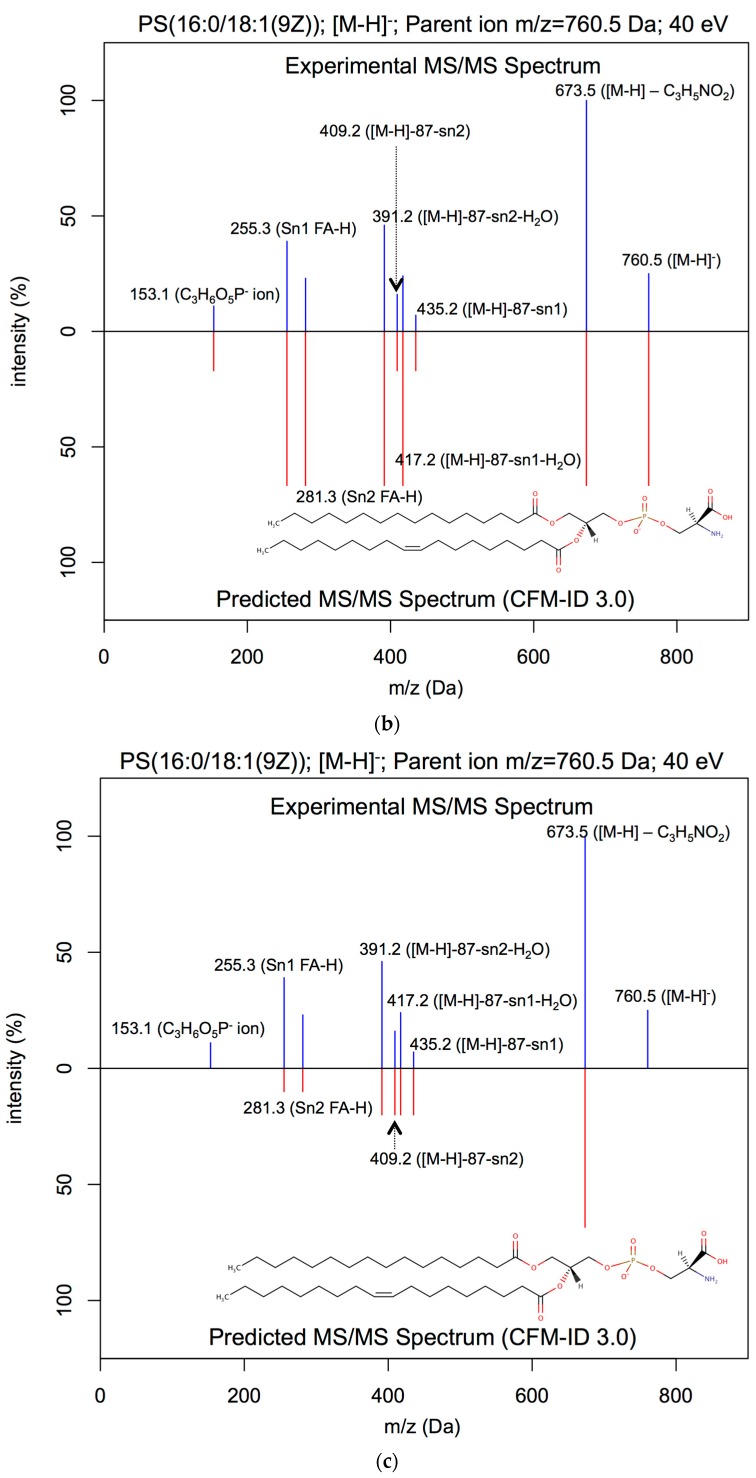
Head-to-tail plot of experimental and predicted ESI-MS/MS spectra of (PS(16:0/18:1(9Z))). (**a**) Head-to-tail plot showing an experimental of ESI-MS/MS spectrum of 1-palmitoyl-2-oleoyl-sn-glycero-3-phospho-L-serine (PS(16:0/18:1(9Z))) measured at 40 eV, and the matching ESI-MS/MS spectrum predicted by CFM-ID 2.0. The computed spectral similarity score is 0.10. (**b**) Head-to-tail plot showing an experimental ESI-MS/MS spectrum of 1-palmitoyl-2-oleoyl-sn-glycero-3-phospho-L-serine (PS(16:0/18:1(9Z))) measured at 40 eV, and the matching ESI-MS/MS spectrum predicted by CFM-ID 3.0. The computed similarity score is 0.92. (**c**) Head-to-tail plot showing an experimental ESI-MS/MS spectrum of 1-palmitoyl-2-oleoyl-sn-glycero-3-phospho-L-serine (PS(16:0/18:1(9Z))) measured at 40 eV, and the matching ESI-MS/MS spectrum predicted by LipidBlast. The computed similarity score is 0.91.

As seen in Figure 2, the spectral similarity between the CFM-ID 2.0-generated spectrum and the experimental ESI-MS/MS spectrum was 0.07, with CFM-ID 2.0 being able to predict only two fragments that were observed in the experimental spectrum (namely, the C5H12N+ and C5H14NO4P+ ion fragments). For this particular example, CFM-ID 2.0 predicted 31 fragments (Figure 2a) while CFM-ID 3.0 predicted 10 fragments (Figure 2b), 7 of which were observed in the experimental ESI-MS/MS spectrum. It is worth noting that the remaining three fragments result from fragmentations that were observed in experimentally measured ESI-MS/MS spectra of phosphatidylcholines obtained for [M+H]^+^ adducts at 40 eV. For this example, the spectral similarity score was 0.88 when comparing the experimental ESI-MS/MS spectrum with the CFM-ID 3.0-predicted spectrum. Surprisingly, the dot product score was only 0.13 when compared with the LipidBlast-predicted ESI-MS/MS spectrum to the experimental ESI-MS/MS spectrum. Figure 3 shows comparisons between experimental and predicted ESI-MS/MS spectra for 1-palmitoyl-2-oleoyl-sn-glycero-3-phospho-L-serine (PS(16:0/18:1(9Z))) in the negative ([M−H]^−^) ion mode at a collision energy of 40 eV. The measured spectral similarity scores between the experimental and the in silico-generated spectra are 0.10, 0.92, and 0.91 with CFM-ID 2.0 (Figure 3a), CFM-ID 3.0 (Figure 3b), and LipidBlast (Figure 3c), respectively.

As highlighted in Table 3, CFM-ID 3.0 significantly outperforms CFM-ID 2.0 in terms of lipid spectral prediction performance (average score of 0.85 versus 0.09) and CFM-ID 3.0 generally outperforms LipidBlast (average score of 0.85 versus 0.34). Another important advantage of CFM-ID 3.0 over LipidBlast is the fact that it generates spectral predictions for multiple collision energies (10, 20, and 40 eV) whereas LipidBlast only provides a single spectrum at an undefined collision energy. Furthermore, all spectral predictions generated by CFM-ID 3.0 include information about not only the *m*/*z* values and their relative intensities but also the structure of the predicted fragments (expressed as InChI and SMILES strings) for every predicted peak. LipidBlast only provides the *m*/*z* values and intensities.

#### 2.3.2. Compound Identification Using the New Scoring Functions

A set of 1,000 compounds was used to train a new and improved scoring function for ESI-MS/MS-based compound identification (see Section 4.5). This function was developed in order to optimize CFM-ID 3.0’s compound identification performance using spectral matching scores, compound classification information from high-scoring hits, and compound metadata (citations). The models were obtained using 5X cross-validation, and tested on different sets. Table 4 compares the performance of CFM-ID 3.0 versus CFM-ID 2.0 (2016 and 2019) and MS-FINDER [26] for compound identification based on 208 ESI-MS/MS spectra from 185 unique compounds. The test involving CFM-ID 2.0 for 2016 and 2019 used the same algorithm and scoring functions. However, the 2019 version mentioned here uses the expanded spectral library, compared to the in silico spectral library of 2016. The test spectra correspond to those provided for the CASMI 2016 contest (category 3). To establish a baseline and ensure the spectral similarity matching method worked, we first queried the 208 experimental spectra against the full spectral database (with those same 208 spectra included). In this case, both CFM-ID 2.0-2019 and CFM-ID 3.0 correctly identified all 208 query compounds with perfect spectral similarity scores. The 208 experimental spectra were then removed from the CFM-ID 3.0’s spectral library and the queries were run again. As illustrated in Table 4, CFM-ID 3.0 was able to correctly identify the query compound in 149 out of 208 challenges, compared to only 123 by CFM-ID 2.0-2019 or 120 by CFM-ID 2.0-2016. This represents an improvement of 21.1% over CFM-ID 2.0. The query compound was generally ranked higher (average rank = 1.8) by CFM-ID 3.0 compared to CFM-ID 2.0-2019 (average rank = 2.4). CFM-ID 3.0 also achieved a better medal score (848) compared to CFM-ID 2.0-2019 (718). A medal score is calculated as the sum of weighted top 1 ranks with 5 points (gold medal), top 2 ranks with 3 points (silver), and top 3 ranks (bronze) with 1 [19]. CFM-ID 2.0-2016 and MS-FINDER [26] were also evaluated in the CASMI 2016 contest (category 3). As the original winner of the CASMI 2016 contest, MS-FINDER correctly identified the query compound in 146 cases [19]. However, as shown in Table 4, MS-FINDER scored 20% fewer top 3 hits compared to CFM-ID 3.0. Moreover, MS-FINDER achieved a lower medal score (766 versus 848), and had a much lower average “hit” rank (6.4 versus 1.8) compared to CFM-ID 3.0. It is also worth noting that CFM-ID 2.0-2016 scored the lowest in terms of top 1 hits, had the lowest average “hit” rank (13.6), and the lowest medal score (just 600).

#### 2.3.3. Compound Chemical Classification

For this assessment, the 208 challenge MS/MS spectra (corresponding to 185 distinct compounds) from the 2016 CASMI competition were used as queries. CFM-ID 3.0 was used to predict the chemical class of the query compound with the predicted class being the direct parent (according to ClassyFire [20]) of the highest-ranked compound. The direct parent is the parental or broader chemical class in the ClassyFire hierarchy to which a compound belongs. It typically corresponds to the largest identifiable chemical skeleton or most dominant feature of the classified compound [20]. In case of a tie, the predicted class was identified as the most frequently occurring chemical class among the direct and alternative parents of all compounds with the highest score. Alternative parents are categories in the ClassyFire ontology that describe the classified compound and do not display a parent–child relationship to each other or to the direct parent [20]. When using ESI-MS/MS spectra as input, CFM-ID 3.0 correctly predicted the chemical class in 168 out of 208 challenges. Interestingly, in 19 out of the 168 challenges, the corresponding query compound could not be correctly identified. These results suggest that CFM-ID 3.0 was still able to capture key structural features that characterize the fragmentations observed in the corresponding input MS/MS spectra. These findings also demonstrate the importance of using a diverse set of compounds and MS/MS spectra to assist with compound identification or classification. In particular, they highlight the need for large compound/spectral databases for proper compound identification.

## 3. Discussion

### 3.1. ESI-MS/MS Lipid Spectra Prediction

The much better performance for lipid spectra prediction via rule-based fragmentation approaches (CFM-ID 3.0) relative to combinatorial fragmentation approaches (CFM-ID 2.0) is likely due to two factors. First, lipids are modular molecules and so the MS fragmentation patterns seen under most collision energies are easily understood and relatively simple to describe. On the other hand, combinatorial fragmenters have no knowledge of molecular structure and so they cannot recognize modular structures. Instead, they view lipids as molecules with dozens of breakable bonds, all of which could potentially be fragmented. This leads to a substantial over-prediction of MS peaks. The second reason why combinatorial fragmenters do not perform well on lipids is that they have generally not been “trained” on lipid spectra. For example, CFM-ID 2.0 was only trained on ~1000 experimental MS/MS spectra, none of which included lipid MS/MS spectra. Similarly, MetFrag [9], another combinatorial fragmenter, was also not originally programmed to handle lipid MS/MS spectra (although a later version was [27]). By expanding CFM-ID’s training set and including lipid spectra as well as other modular compound classes in that training set, CFM-ID could potentially improve its performance to match even the rule-based fragmenter.

Overall, our results show that CFM-ID 3.0 was able to reproduce most lipid fragments with accurate *m*/*z* ratios and reasonably accurate relative intensities. Characteristic fragment ion losses (e.g., loss of polar head, or side chains) were also well reproduced. CFM-ID 3.0’s spectral predictions also include many ion fragments that are independent of the acyl or alkyl chain(s) of the molecular ion, including the cyclic 1,2-cyclic phosphate diester (neutral *m*/*z* = 125.0003) fragment, which is often observed in ESI-MS/MS spectra of various choline glycerophospholipids. Interestingly, most of these kinds of fragments are not reported in LipidBlast-generated spectra. On average, the spectral similarity score between experimental ESI-MS/MS spectra and LipidBlast-generated spectra was 0.34 ± 0.4. One of the reasons for the lower similarity scores for LipidBlast has to do with the fact that it generates only one consensus spectrum per compound, which tends to more closely match with experimental ESI-MS/MS spectra collected at 40 eV. As a result, the average spectral similarities for LipidBlast-generated spectra to experimental spectra obtained at 10 and 20 eV are very low.

As expected, some discrepancies were observed when comparing predicted MS/MS spectra with the corresponding experimental MS/MS spectra. First, the relative peak intensities were generally found to be higher in the predicted MS/MS spectra than the experimental spectra. Second, the peak lists are often not identical. MS/MS spectral peak intensities are very difficult to predict and vary considerably depending on the instrument, the instrument parameters, and experimental design. For instance, phosphatidylcholines, when analyzed by Q-TOF instruments, tend to lose the molecular ion even at medium collision energies. On the other hand, when phosphatidylcholines were analyzed on ion-trap MS instruments, the molecular ion was still highly abundant at medium collision energies, and was significantly fragmented only at high energies [28,29,30]. In addition to instrument differences, the type of solvent being used can affect the extent to which a compound is fragmented. However, rather than focusing on these subtleties, we chose to focus on selecting (and annotating) the most abundant or most characteristic fragments, which were generally reproducible on different instruments, and reported in multiple studies.

While CFM-ID 2.0 predicts fragmentation probabilities and numeric peak intensities for all query compounds, CFM-ID 3.0 does not predict numeric peak intensities for lipid spectra (however, it still predicts numeric peak intensities for all other classes of molecules). Instead, CFM-ID 3.0 predicts categorical peak intensities for lipid spectra (low, medium, high, and maximum abundance). This simple categorization partly explains why, in many cases, the relative peak intensity is higher in predicted lipid spectra compared to experimental spectra. We believe that a larger lipid MS spectral training set (at least 10+ spectra per chemical class and adduct type) would help to improve the prediction of numeric intensities and simulate their variation between collision energies more accurately. Another limitation of CFM-ID 3.0’s rule-based approach for lipid prediction is that the current fragmentation rules do not take the information about the stereochemistry and the position of double/triple bonds into consideration. Therefore, the existing rules do not allow one to distinguish between stereoisomers or regiomers. This is a common problem for rule-based “fragmenters”, since the incorporation of such distinctions would require the acquisition of a much more diverse and larger set of high-resolution MS^n^ spectra.

CFM-ID 3.0 returns the structure (in InChI or SMILES strings) for all predicted fragments. This helps to provide a rationale for nearly all observed peaks. Additionally, this linkage simplifies the lipid ESI-MS/MS spectral annotation process. Because CFM-ID 3.0 provides MS/MS spectra at three energy levels (10, 20, and 40 eV), it means that the predicted MS/MS spectra can be matched more closely to real experimental conditions and real experimental MS/MS spectra. Many other spectral libraries (LipidBlast, NIST) only provide consensus MS/MS spectra for lipids, which makes it difficult to relate experimental data to the predictions.

### 3.2. Compound Identification and Chemical Class Prediction

The incorporation of citation counts in MS-based compound identification protocols has been consistently shown to improve identification rates in recent studies on spectral/compound identification [19,31]. However, an obvious limitation of this approach is that it reduces the probability of identifying novel or rare compounds that have never been cited. Citation counts can also bias the ranking scheme to select one very similar structure (and therefore a very similar MS spectrum) over another purely on the basis of one having slightly more citations than another. To help balance the influence of citation counts, we incorporated chemical classification into our new scoring system. In this way, the scientific relevance or approximate abundance (in terms of citations) as well as the structural features among candidates could be taken into consideration. Using this approach, a new scoring function was developed for compound identification in CFM-ID 3.0. This new function helped to improve MS-based compound identification quite significantly (see Table 4). When applied to 208 identification challenges on a CFM-ID spectral library containing the 208 ESI-MS/MS spectra, both CFM-ID 2.0 and CFM-ID 3.0 were able to identify all 208 compounds based on spectral similarity. However, as pointed out earlier, it can be expected that most spectral libraries, including CFM-ID 3.0’s, will not include (the same) experimental spectra corresponding to a compound of interest. Thus, the use of metadata (i.e., citations) in addition to spectral similarity can help improve identification rates. In particular, when applied to 208 identification challenges, CFM-ID 3.0’s ESI-MS/MS scoring function achieved an improvement in overall ranking and identification rate (21.1%) over CFM-ID 2.0’s original scoring function. We believe the use of diverse training sets of compounds, representing widely varying structures and structural classes was critical to achieving this improved performance. Our work in this area also confirmed the notion that more work needs to go into expanding spectral databases with experimental data and that this will ultimately improve spectral/compound identification performance.

CFM-ID 3.0 was also assessed with regard to its performance in chemical class prediction. While it may not be possible to identify the exact compound via MS/MS spectral matching, the ability to use MS/MS spectra to narrow down the correct chemical class or chemical family for a given query spectrum or compound can be very valuable for many applications in metabolomics or natural product de-replication. In assessing the performance of CFM-ID’s chemical class prediction, the same scoring system introduced here was used to rank the individual candidates. However, in order to perform a formal chemical class identification, the query compound was predicted to belong to the “direct parent” class of the highest-ranked candidate. In cases of a tie, the predicted chemical class was predicted to be the most frequently occurring among all the direct and alternative parents among all the compounds with the highest score. Upon testing the new ESI-MS/MS scoring function on 208 challenges, the correct class was predicted in 80.8% of the challenges (compared to 71.6% for correct compound identification). This result indicates that even when a compound was not identified correctly, the correct class prediction could still be made (in 19 cases). This suggests that CFM-ID 3.0 is still able to identify structural features that characterize the MS/MS fragmentation patterns of certain classes of compounds. These results also demonstrate the importance of using a diverse set of compounds and spectra, as well as the need of having a sufficiently large database to enable compound (or compound class) identification via spectral matching. Structurally similar compounds tend to produce similar spectra. Therefore, even if the compound (and its corresponding MS/MS spectrum) is not in the database (or is poorly ranked), a large number of compounds/spectra from related compound classes could still help to identify the correct compound class. We believe that this helped CFM-ID 3.0 achieve its relatively good performance in the class prediction task.

The inclusion of additional data (citation frequency and chemical class information) in the CFM-ID scoring functions clearly improved compound identification performance. We also believe these improvements were partially aided by the much-improved quality of lipid MS/MS spectra predicted by CFM-ID 3.0. While we made substantive improvements to the quality of CFM-ID’s lipid spectra prediction, more work still needs to be done in CFM-ID to better predict the MS/MS fragmentation of other classes of compounds (such as alkaloids, polyphenols, terpenes, and steroids) and to increase the quality of other predicted MS/MS spectra. The addition of many more experimental ESI-MS/MS spectra, measured with various MS instruments, and under different conditions, is also expected to help capture spectral patterns that are not yet accurately predicted by CFM-ID’s algorithm, and thus, improve its overall compound identification performance.

## 4. Materials and Methods

To improve CFM-ID’s overall performance for MS/MS analysis, we pursued several algorithmic and database enhancements such as (1) encoding and validating rules for ESI-induced fragmentation of 21 classes of lipids; (2) implementing an automated chemical classification schema (via ClassyFire) for both CFM-ID’s database and its query compounds; (3) redesigning, expanding, and improving CFM-ID’s MS/MS spectral library (by including experimental MS/MS spectra and adding many thousands of predicted ESI-MS/MS spectra, as well as metadata); (4) collecting citation information from various sources for all of the compounds in CFM-ID’s MS/MS spectral library; and (5) modifying CFM-ID’s scoring function to incorporate the above changes and improve its overall performance.

The encoding of the lipid rule-based fragmentation approaches was added to improve the speed and accuracy of CFM-ID’s lipid ESI-MS/MS predictions, as well as to cover a larger pool of experimental conditions as reflected by different adduct types. The use of ClassyFire’s chemical classification method [20] was implemented to automate the rule-based/combinatorial-based decisions for CFM-ID and to improve CFM-ID’s ability to identify or re-rank potential MS/MS spectral matches based on structural similarity. The redesign and expansion of the CFM-ID’s spectral database was performed to accelerate search speeds, reduce the memory requirements, and to grow the spectral database size (of both predicted and known MS/MS spectra) by a factor of 2.6 so as to improve the likelihood of user query spectral matches. The inclusion of citation data was intended to enhance the scoring accuracy of potential MS/MS spectral matches, while the modification of CFM-ID’s scoring function was intended to improve its overall performance. Details regarding how all of these changes were implemented are described below.

### 4.1. Encoding Lipid Fragmentation Rules

Our analysis of numerous databases and the literature indicated that there are 21 major classes of lipids for which MS/MS spectra are best predicted using hand-made fragmentation rules. The encoding of these hand-made lipid fragmentation rules involved several steps: (1) experimentally measuring or compiling (via literature) characteristic MS/MS fragment ions observed at each of three collision energy levels (10, 20, and 40 eV) for each lipid class, (2) determining the relative abundance of each fragment ion at each energy level, (3) accurately determining the chemical structure and *m*/*z* values of each of the fragment ions, (4) including more MS/MS experimental conditions (and adduct ions) by expanding the list of adduct types covered by previous versions of CFM-ID, and (5) implementing these rules using standardized cheminformatics languages (SMARTS [32] and SMIRKS [33]) in order to rapidly and accurately predict and annotate ESI-MS/MS spectra for lipids.

#### 4.1.1. Acquisition of Reference Lipid MS/MS Spectra

The generation of the lipid fragmentation rules required the acquisition of experimental ESI-MS/MS spectra for a number of lipids and lipid classes. The acquired spectra were collected at several collision energies, for various adduct types (e.g., [M+H]^+^, [M−H]^−^), and, if possible, from various MS instruments. This was used to help capture fluctuations or biases that can be introduced by the different parameters. A total of 533 experimental MS/MS spectra were collected for 16 standard lipids (purchased from Avanti Polar Lipids Alabaster, AL) from 15 lipid classes at various collision energies (10 to 60 eV), in both positive and negative mode using an AB Sciex QTrap 4000 MS instrument. For each lipid standard, an enhanced MS (EMS) scan was first collected to identify precursor ions with high abundance in either ionization mode. Enhanced product ion (EPI) scans were then collected for each precursor ion to generate the MS/MS spectra with different collision energy levels ranging from 10 to 60 eV, with the supervision of a mass spectrometry expert. For more information about the collection of spectra for the 16 standard lipids, see Supplementary Material. In addition to the MS/MS spectra collected in our laboratory, published lipid MS/MS spectral data were compiled from the LIPID MAPS [28] and the MoNA [24] databases. For the LIPID MAPS spectra, only annotated spectral images were available. Therefore, MS/MS peak lists were generated by annotating the peaks using a semi-automated approach. This approach consisted of computing the relative abundance of each peak, and manually mapping it to the *m*/*z* list provided in the LIPID MAPS spectrum. In addition to the experimental spectra, the LipidBlast and FAHFA 26 libraries, as well as MassBank [34], mzCloud [35], and the sphingolipid library of Tsugawa et al. [18] served as references that provided additional information for lipid classes not covered by our experiments. In total, 844 lipid MS/MS spectra from 21 lipid classes were collected and analyzed.

#### 4.1.2. Annotation of Reference Lipid MS/MS Spectra

With the lipid MS/MS spectra in hand, we proceeded to manually annotate each spectrum. This consisted of assigning each fragment ion peak to a specific structure and a specific reaction or fragmentation event (e.g., the loss of a water molecule from a [M+H]^+^ precursor ion, the loss of a side chain, or the presence of a specific fragment). The annotation of spectra was limited to the in-house generated MS/MS spectra and the LIPID MAPS set, as both were measured with the same model of instrument (AB Sciex QTrap 4000). The annotation process was largely guided by the information provided in LIPID MAPS, LipidBlast, and other scientific reports [17,28,29,36]. In a number of cases, the same compound had MS/MS spectra in at least two of the data sets (including the LipidBlast database), and the corresponding spectra were available for the same adducts or ions. In these cases, we annotated the spectra by direct comparison of the peak lists. Among the 21 lipid classes, 11 were not covered by our in-house experimental data. For this reason, the MS/MS spectra of these missing lipid classes were extracted from the LIPID MAPS (experimental) and/or LipidBlast (in silico) library. Since the experimental and theoretical spectra acquired from other sources (LipidBlast, LIPID MAPS) did not always cover all three collision energy levels (10, 20, and 40 eV), the generation of consensus fragmentation patterns was done by comparing standards to corresponding experimental spectra obtained under the same conditions (adduct type and collision energy). Moreover, when applicable, experimental MS/MS spectra collected from other sources (e.g., MoNA) and obtained under similar experimental conditions were compared to one another, as well as to theoretical spectra. In particular, theoretical spectra from LipidBlast helped in the spectral annotation. The fragmentation and spectral annotation rules were further validated by mining the scientific literature and acquiring/confirming additional spectral data from published papers. Once the energy-specific fragmentation patterns were generated, the relative abundance of each peak was assigned to one of the four intensity levels: low (1–15%), medium (15–60%), high (60–90%), or maximum (90–100%) abundance level. The assigned intensity was based on observed relative abundances from our experimental spectra. The maximum level of abundance was assigned to the base peak, typically when no fragmentation was observed (usually at a low collision energy). Additional feedback from local MS experts combined with an extensive review of the lipid MS/MS literature helped to complete the spectral annotation process. This effort led to the near-complete annotation of all observed fragment ions, their precise *m*/*z* values, and the corresponding fragmentation reactions for a total of 610 peaks from 21 lipid classes at each of 3 collision energies (10, 20, and 40 eV).

#### 4.1.3. Implementation of Lipid Fragmentation Rules

The annotated fragment ions along with their structures and reactions provided the basis for the creation of fragmentation rules. All of the fragmentation rules were implemented in the Java programming language through a new “lipid fragmenter module” in CFM-ID. The structural backbone of each lipid or lipid fragment class was represented using the Daylight SMARTS language [32]. This is a module implemented in ClassyFire (version 2.1), a software tool for automating structure-based hierarchical annotation of chemicals [20]. To accelerate the lipid classification process, a sub-ontology from the ChemOnt [20] ontology was used. For each lipid or lipid fragment class, one set of fragmentation patterns is encoded for each of the applicable adducts as chemical reactions. The chemical reactions are represented using the Daylight SMIRKS language [33]. Moreover, SMARTS strings are used to select the appropriate fragments [32]. Additionally, a number of transformation rules were encoded to standardize the structures of all the query compounds. The standardization of the fragmentation reactions using well-developed cheminformatics languages ensures that the structural representations are consistent for all query compounds, structural classes, and chemical reactions. Without adhering to these standards many chemicals classes could be misidentified or invalid fragments could be returned.

The new CFM-ID lipid fragmenter program has been fully integrated into the existing spectral prediction workflow of the previous version of CFM-ID [12]. In CFM-ID 3.0, the lipid MS/MS prediction tasks require a lipid structure (submitted as a SMILES string or SDF file) and an adduct or an ion as input. Upon submission, the compound is classified based on its structure via ClassyFire. If the compound is identified by ClassyFire as a lipid molecule belonging to any of the covered classes and if the fragmentation patterns applicable to the selected adduct exist in the lipid fragmentation library, then the compound is fragmented accordingly. The fragmentation operation is executed using the AMBIT library [37]. After the in silico fragmentation step is completed, the relative abundance of each peak is assigned (using the fragmentation rules described above), and three ESI-MS/MS spectra are generated (at 10, 20, and 40 eV). Relative intensities are assigned using a set of pre-calculated intensities based on the chemical class, the adduct type, and the collision energy. The fragmentation patterns, as well as the relative intensities of the resulting peaks, are the same for all compounds from the same chemical class, under the same experimental conditions (i.e., adduct type and collision energy). If no set of fragmentation patterns is applicable to the compound and/or the selected adduct, and the compound is not a glycero-, phospho- or sphingolipid, then the ESI-MS/MS spectra are predicted using the original CFM-ID algorithm as implemented in CFM-ID 2.0. However, if the compound is a glycero-, phospho- or sphingolipid, the computation is stopped, and an error message is returned. This is done to ensure that CFM-ID does not use the combinatorial fragmentation algorithm, which, as mentioned, does not perform well for such compounds. The resulting ESI-MS/MS spectra are then returned with each peak annotated by its *m*/*z* value, its relative abundance, and the chemical structure of the corresponding fragment encoded in a standard SMILES format. Additionally, any available experimental MS spectra in the CFM-ID spectral database matching the query compound are also displayed in the results alongside the predicted spectra.

### 4.2. Integration of Chemical Classification

Similar structures tend to undergo similar MS fragmentation events under the same conditions. For this reason, a number of in silico MS fragmentation algorithms now take the chemical structure of query molecules into consideration for improved MS-spectra prediction and compound identification tasks. For the prediction of EI-MS/MS spectra, CFM’s scoring function partly relies on a list that describes the presence or absence of 107 functional groups and 86 fragment descriptors. These groups and fragment descriptors are provided by ClassyFire [20] and RDKit [38], respectively. Other computational tools such as CSI:FingerID [7] rely on models that can predict the presence of functional groups and fragments based on a given compound or a given MS-spectrum. For this reason, it might be expected that for compound identification tasks, the highest-ranked candidates would likely share a significant number of functional groups or possibly share a maximum common substructure. This information would be particularly helpful in cases where it is very difficult to discriminate between the highest-ranked candidates. More specifically, the presence of one or more common structural backbones (e.g., diterpene, ceramide, phosphatidylglycerol, etc.) could significantly impact the ranking, when very structurally similar candidates are prioritized among those that have a high spectral similarity to the query compound.

Therefore, a chemical classification was assigned to each compound in CFM-ID 3.0’s database. The chemical classification was computed by ClassyFire and retrieved using the ClassyFire API [20]. As will be described later in this section, the chemical class assigned to candidate molecules was taken into account along with other metadata to improve the original CFM scoring method (dot product or Jaccard score). In addition to the adjustment of the scoring function, chemical classification was also used to predict the chemical class(es) to which the query compound belonged. Formally, the predicted chemical class corresponds to the direct parent of the highest-ranked candidate. In case of a tie, the predicted chemical category is the most frequently occurring direct or alternative parent among all candidates that has the highest score.

### 4.3. Collection of Compound Citations

Several studies have demonstrated that the integration of metadata can significantly improve compound identification rates with spectral library searches [7,9,19]. In particular, the frequency with which a compound is mentioned in the literature could serve as a proxy for the likelihood that the compound is sufficiently abundant for detection via MS/MS methods. Therefore, every compound in the CFM-ID spectral library was assigned a citation score. An initial set of citation counts was obtained using DataWrangler. DataWrangler is an in-house tool that automatically mines PubChem [39], HMDB [15], ChemSpider [40], and ChEBI [41], and returns a unique list of scientific reference citations for a given compound. A second set containing PubMed citation counts (without PubMed IDs) was obtained by mining the EPA’s CompTox dashboard [42]. This set was computed and generously provided to us by the CompTox dashboard’s development team. The two sets were merged by comparing each compound’s InChI keys. More specifically, when a compound had a citation count in only one set, the corresponding citation count was assigned to that compound. For compounds that had citation counts both from DataWrangler and CompTox, the largest count was assigned, as it was expected that both counts could include many of the same citations. A total of 140,379 compounds were assigned a citation count of 1 or more. For the remaining compounds, DataWrangler assigned a custom citation count of 1, if and only if, they were found in at least one of the following databases: HMDB [15], DrugBank [21], T3DB [43], ContaminantDB [44], FooDB [45], ECMDB [46], YMDB [47], and PhytoHub [22].

### 4.4. Redesigning and Expanding of CFM-ID’s Spectral Library

The original reference spectral library in CFM-ID 2.0 contained unique computationally generated ESI-MS/MS spectra for ~51,000 compounds from the HMDB and KEGG databases. These in silico ESI-MS/MS spectra were computed in positive ([M+H]^+^) and negative ([M−H]^−^) ionization modes, one for each of three collision energies (10, 20, and 40 eV). In order to significantly improve identification rates, the new CFM-ID library was updated as described below.

#### 4.4.1. Collection of Experimental MS/MS Spectra from External Sources

While the accuracy of computationally predicted MS spectra is often quite good, the accuracy of experimentally collected MS spectra is obviously much better. Therefore, the inclusion of experimentally determined ESI-MS/MS spectra would be expected to improve the match scores for query spectra/compounds that have previously been analyzed by ESI-MS/MS. Experimentally determined ESI-MS/MS spectra were downloaded from the MassBank of North America’s (MoNA) online repository [24]. As of February 2019, MoNA contained 89,861 experimental LC-MS/MS spectra for 12,799 compounds. The spectra and compounds in MoNA originate from several databases, including the HMDB database [15], MassBank [18], the GNPS database [23], and the ReSpect database [48], among others. Only experimental spectra were collected from MoNA. An additional 915 ESI-MS/MS spectra were manually regenerated for 523 compounds from information contained in the NIST 14 database.

Since CFM-ID uses models trained on MS/MS spectral data sets that use specific collision energy and mass accuracy criteria, the HMDB, MoNA, and NIST spectra were further filtered to match these criteria. Specifically, experimental MS spectra were required to have a known ionization type, a known compound neutral mass, and to have been analyzed with high-resolution MS instruments (e.g., Q-TOF instruments) in the case of LC-MS spectra. After filtering, there were 72,678 usable experimental MS/MS spectra remaining from the MoNA dataset. These experimental MS/MS spectra were converted into the peak list format required for CFM-ID and uploaded into CFM-ID’s online spectral library. In addition, the complete library of experimental MS/MS spectra from the HMDB was obtained from our in-house repository, and filtered. Upon filtering, this library contained 1152 unique ESI-MS/MS spectra for 239 unique compounds.

#### 4.4.2. Compilation of Predicted ESI-MS/MS Spectra

The original CFM-ID 2.0 database contained 102,153 unique computationally generated ESI-MS/MS spectra (from 51,635 compounds). Among the 102,153 ESI-MS/MS spectra, 36,746 were previously computed for 18,373 unique compounds belonging to the 21 lipid classes covered by the rule-based fragmenter, and transferred to the CFM-ID 3.0 database. The remaining 65,407 mass spectra computed by CFM-ID 2.0 were also moved to the CFM-ID 3.0 database. In total, ~36,900 spectra were generated for 18,438 lipids. To this database, another ~207,956 ESI-MS/MS spectra were computed for another ~80,000 lipids and 7288 other metabolites obtained from new versions of HMDB, DrugBank, and PhytoHub. These compounds were added to the CFM-ID 3.0 database. These predicted ESI-MS/MS spectra were generated for both positive and negative ion mode as well as at three different collision energies (10, 20, and 40 eV). In total, the CFM-ID 3.0 database now contains 310,109 computationally generated ESI-MS/MS spectra (from 155,544 compounds). If the experimental ESI-MS/MS spectra are added to this total, the CFM-ID 3.0 spectral database now contains a grand total of 393,158 ESI-MS/MS spectra.

### 4.5. Modifying CFM-ID’s Scoring Function and Ranking Schema

The results of the Critical Assessment of Small Molecular Identification (CASMI) 2016 contest showed that the integration of additional data (i.e., citation frequency of compounds and structure similarity) into the original scoring function for CFM-ID improved compound identification rates [19]. This trend was also observed for several other tools during the contest and in separate studies [9,19]. To create a combined score, the original dot product spectral similarity score computed by CFM-ID was combined with a citation score and a chemical classification score. The citation score is based on the number of citations that a given compound has in the scientific literature. More highly cited compounds are typically those that are more commonly detected, studied, or used. Therefore, the citation score serves as a proxy of the general abundance or concentration of a compound and is intended to favor more abundant compounds over extremely rare or compounds at very low biological concentrations.

The chemical classification score is based on the number of chemical categories to which a compound is assigned (by ClassyFire), relative to the total pool of chemical classes assigned to all candidate molecules. The chemical classification score was added to help re-rank or cluster structurally similar molecules (and MS spectra) closer together. Each of the three scores was normalized by dividing its computed score by the maximum score across the candidate list. The general formula for the total candidate score is:

$$S_{TOTAL}\left(C\right)=a_{CFM_{ORIG}}*S_{CFM_{ORIG}}\left(C\right)+a_{CLASS}*S_{CLASS}\left(C\right)+\;a_{REF}*S_{REF}\left(C\right)$$

where *S_TOTAL_*(*C*), *S_CFM_ORIG_*(*C*), *S_CLASS_*(*C*), and *S_REF_*(*C*) are the total score, the normalized spectral matching CFM-ID score, the normalized ClassyFire score, and the normalized reference score for candidate *C*, respectively. Each of the three scores are weighted by the coefficients a*_CFM_ORIG_*, a*_CLAS_S__*, and a*_REF_*, respectively, where:

$$a_{CFM\_ORIG},\;a_{CLASS},\;a_{REF}\geq 0$$

and

$$a_{CFM\_ORIG}+a_{CLASS}+a_{REF}=1$$

This scoring function was then iteratively optimized on a training data set to maximize its metabolite identification potential. In particular, the optimal set of coefficients was determined through a grid search using a manually selected set of 1000 spectral/compound identification tasks (for 1000 unique compounds ranging from drugs to lipids). Each of the selected molecules had one or more experimental spectra at one of three level energies (10, 20, and 40 eV), in addition to the predicted ESI-MS/MS spectra. The data set was divided into five equally sized subsets. Several models (with a unique combination of coefficients) were trained on 800 compounds (4/5 of the data set) and tested on the remaining 200 (1/5 of the data set). This process was repeated four more times, using a different test set of 200 compounds for each iteration. Experimental spectra were used as input for each identification test, and upon testing, only the best model was selected. A consensus model was built based on the five selected models, and further tested using a smaller test set. The final coefficient values for the ESI-MS/MS scoring function were a*_CFM_ORIG_* = 0.6, a*_CLAS_S__* = 0.1, and a*_REF_* = 0.3.

This function was further refined to improve its performance and to deal with certain extreme or rare situations. In particular, we observed certain cases in which the spectral similarity between a query and a database match is so close that applying the citation and chemical classification scores causes such strong matches to be unfairly penalized. In order to prevent this, we implemented a 95% similarity threshold, above which only the original spectral similarity score is applied, and the citation and classification information is disregarded. Moreover, our training showed that the enormous discrepancy between citation counts (from 2 to >30,000) could negatively impact the identification rate. For instance, a compound that would be correctly identified using spectral similarity (with or without metadata) could be easily ranked much lower, in favor of a similar compound with a significantly high citation score. Thus, the citation count was adjusted to have a ceiling corresponding to 156 (2 × average citation count) for every compound that has over 156 citations.

### 4.6. Performance Testing

Three types of performance tests were conducted. The first assessed the performance of the lipid ESI-MS/MS spectral prediction method, the second assessed the performance of the new scoring function in exact compound identification, and the third assessed CFM-ID’s performance in identifying a compound’s correct chemical class. To test the lipid ESI-MS/MS spectral prediction method, a benchmark analysis was performed on 20 randomly chosen lipids from each of the 21 known lipid classes for which fragmentation rules were derived. The computation was performed on a 2.7 GHz Intel Core i5 running macOS with 16 GB (1867 MHz DDR3) of memory. A total of 120 ESI-MS/MS spectral predictions were generated for both CFM-ID 2.0 and CFM-ID 3.0 at 3 different energies and 2 different ionization modes with various adduct types. The average execution time was determined for each spectral prediction. In addition to the execution time comparison, an additional performance comparison was conducted to assess the quality of the predicted MS/MS spectra. For this task, a set of 5 experimental ESI-MS/MS spectra measured in positive ion mode, and 9 experimental ESI-MS/MS spectra measured in negative ion mode were selected. The selected spectra were measured under conditions that can be simulated by CFM-ID 3.0’s lipid fragmentation rules (same energy levels, same adducts). For each experimental MS/MS spectrum, CFM-ID 2.0 and CFM-ID 3.0 were used to predict a corresponding MS/MS spectrum under the same conditions. The performance was assessed by measuring the average pairwise spectral similarity between experimental and predicted spectra using a standard dot product score as implemented in the OrgMassSpecR package [49]. Moreover, they were also compared to LipidBlast, as the selected lipids and corresponding predicted spectra were also contained in the LipidBlast library.

The ESI-MS/MS scoring function was tested on a set of 208 experimental ESI-MS/MS spectra (for 185 unique compounds) generated on a Q Exactive Plus Orbitrap (Thermo Scientific), and used for the CASMI 2016 contest (Category 3) [19]. These spectra were used as input for compound identification. One-hundred and twelve of the 185 compounds were included in the database and had at least one experimental ESI-MS/MS spectrum in addition to the pre-computed ones. For each of the remaining compounds, ESI-MS/MS spectra were predicted using CFM-ID and stored in the spectral library. To assess the performance, we used CFM-ID 2.0 and CFM-ID 3.0 scoring functions, separately, to attempt to identify the query compounds. The evaluation was performed in two steps. The first consisted of identifying compounds by searching them in the CFM-ID spectral library that included the 208 experimental ESI-MS/MS spectra from the CASMI. In a second step, the search was performed after excluding the 208 experimentally acquired spectra in the searchable portion of the database. The required mapping between OrbiTrap collision energies and Q-TOF collision energies (which are used by CFM-ID) is described in the Supplementary Material.

For the third assessment, CFM-ID 3.0 was evaluated on its performance in chemical class prediction/identification. This particular task assessment was included because in many situations involving MS-based metabolomics or MS-based natural product identification, it may not be possible to identify the exact compound via MS/MS spectral matching. Therefore, the ability to use MS/MS spectra to reduce the candidate list and to predict the correct chemical class or correct chemical family for a given query spectrum or compound can be very valuable. In assessing the performance of CFM-ID’s chemical class prediction, the query compound was predicted to belong to the “direct parent” class of the highest-ranked candidate. In cases of a tie, the chemical class was predicted to be the most frequently occurring among all the direct and alternative parents among all the compounds with the highest score.

## 5. Conclusions

We have shown that it is possible to substantially improve CFM-ID’s performance in both spectral prediction and compound identification tasks. This was achieved through a number of ways including (1) integrating a rule-based fragmentation approach that currently applies 344 manually curated rules to predict the ESI-MS/MS spectra for 21 classes of common, biologically important lipids, (2) modifying the structure of CFM-ID’s spectral database, and increasing its size by a factor of 2.6, (3) designing new scoring functions that take into account both compound citation frequency and chemical classification features of candidate molecules, and (4) implementing a chemical classification algorithm based on spectral similarity.

In particular, the implementation of a rule-based approach for fragment ion prediction was shown to improve the speed by a factor of 200X and the accuracy of the lipid ESI-MS/MS spectra prediction by a factor of 10X. The success of using rule-based fragmentation patterns encoded in standard chemical representations (SMILES, SMARTS, and SMIRKS) suggests that this concept could be successfully applied to other classes of modular molecules such as carnitines, polyphenols, terpenes, and carbohydrates. The construction and expansion of CFM-ID’s spectral library has also helped CFM-ID’s overall performance. The most recent spectral library has been expanded by a factor of 2.6 over the previously released spectral library. This expansion process is still ongoing, and we plan to include ~500,000 more compounds including drugs, lipids, environmental pollutants, phytochemicals, food compounds, as well as their predicted metabolites generated by BioTransformer [50]. The new scoring function, which already showed an improvement over CFM-ID 2.0’s scoring function, could potentially be further improved by using machine learning techniques and training over a much larger set of MS/MS spectra. Moreover, the acquisition and incorporation of other metadata, such as retention time or collisional cross section information, could help further increase the compound identification rates, as demonstrated in several recent studies [9,19].

The fields of in silico metabolomics and in silico mass spectrometry are rapidly evolving. Thanks to the many excellent ideas emerging in many labs around the world and the willingness of many researchers to share their code and their databases, it is likely that these fields will continue to grow and continue to inspire others to make MS spectral analysis, MS spectral prediction, and MS-based compound identification better, faster, and even more informative.

## Figures and Tables

**Table 1:** Number of fragmentation rules and adduct types covered for each chemical category.

Lipid Class	Number of Covered Rules	Number of Covered Adduct Types
1-Monoacylglycerols	8	[M+Li]^+^; [M+NH4]^+^
2-Monoacylglycerols	11	[M+H]^+^; [M+NH4]^+^; [M+Na]^+^
1,2-Diacylglycerols	10	[M+NH4]^+^; [M+Na]^+^
Triacylglycerols	19	[M+Na]^+^; [M+NH4]^+^; [M+Li]^+^
Phosphatidic acids	22	[M+H]^+^; [M+Na]^+^; [M−H]^−^
Phosphatidylcholines	41	[M+H]^+^; [M+Na]^+^; [M+Li]^+^; [M+Cl]^−^
Phosphatidylethanolamines	24	[M+H]^+^; [M+Na]^+^; [M−H]^−^
Lysophosphatidylcholines	29	[M+H]^+^; [M+Na]^+^; [M+Li]^+^; [M+Cl]^−^
Lysophosphatidic acids	12	[M+H]^+^; [M−H]^−^
Phosphatidylserines	28	[M+H]^+^; [M+Li]^+^; [M+Na]^+^; [M−H]^−^
Ceramides	17	[M+H]^+^; [M+Li]^+^; [M−H]^−^
Sphingomyelins	13	[M+H]^+^; [M+Li]^+^; [M+Na]^+^
Cardiolipins	13	[M−2H](2H)^−^
Phosphatidylglycerols	11	[M−H]^−^
Lysophosphatidylglycerols	7	[M−H]^−^
Plasmanyl-PC (1-alkyl,2-acylglycero-3-phosphocholines)	17	[M+H]^+^; [M+Cl]^−^
Plasmenyl-PC (1-(1Z-alkenyl)-glycero-3-phosphocholines)	17	[M+H]^+^; [M+Cl]^−^
1-Alkanylglycerophosphocholines (Monoalkylglycerophosphocholines)	15	[M+H]^+^; [M+Cl]^−^; [M+Na]^+^
1-Alkenylglycerophosphocholines (1-(1Z-alkenyl)-glycero-3-phosphocholines)	13	[M+H]^+^; [M+Cl]^−^
Phosphatidylinositols	9	[M−H]^−^
Lysophosphatidylinositols	8	[M−H]^−^
Total	344	50

**Table 2:** Statistics for the Competitive Fragmentation Modeling-ID (CFM-ID) 3.0 spectral database.

Feature	Value
Total number of unique compounds	229,084
Total number of unique ESI-MS/MS spectra	397,679
Total number of experimental ESI-MS/MS spectra	87,570
Total number of predicted ESI-MS/MS spectra	310,109
Number of compounds with ≥1 experimental ESI-MS/MS spectra	13,537
Number of compounds with ≥1 predicted ESI-MS/MS spectra	108,972
Number of compounds with ≥2 citations	229,084
Average number of citations per compound	272
Number of compounds with chemical classification assignments	229,084
Average number of chemical category assignments/compound	25

**Table 3:** Computed spectral similarity scores between experimental and predicted ESI-MS/MS spectra at three energy levels (10, 20, and 40 eV). The results show higher similarities, and thus an improvement when using a rule-based approach (CFM-ID 3.0) over a combinatorial one (CFM-ID 2.0) for the prediction of lipid ESI-MS/MS spectra. The spectral similarities of the LipidBlast-generated consensus spectra further illustrate this trend. When available, the same LipidBlast-generated consensus spectrum was used for comparisons at each energy level. N/A corresponds to cases where (1) the adduct type was not covered by CFM-ID 2.0 at all, or (2) the adduct type was not covered by LipidBlast for the chemical class to which the test compound belongs.

Compound	Adduct	Energy (eV)	CFM-ID 3.0 (Score)	CFM-ID 2.0 (Score)	LipidBlast (Score)
PA(16:0/18:1(9Z))	[M−H]^−^	10	1.00	0.36	0.00
PS(16:0/18:1(9Z))	[M−H]^−^	10	1.00	0.31	0.00
CL(18:0/18:0/18:0/18:0)	[M−2H](2H)	10	0.98	N/A	0.00
DG(18:0/20:4/0:0)	[M+Na]^+^	10	0.92	0.00	N/A
PA(16:0/18:1(9Z))	[M−H]^−^	20	0.55	0.02	0.00
PS(16:0/18:1(9Z))	[M−H]^−^	20	0.98	0.03	0.00
CL(18:0/18:0/18:0/18:0)	[M−2H](2H)	20	0.97	N/A	0.12
DG(18:0/20:4/0:0)	[M+Na]^+^	20	0.93	0.00	N/A
PA(16:0/18:1(9Z))	[M−H]^−^	40	0.96	0.03	0.90
PS(16:0/18:1(9Z))	[M−H]^−^	40	0.92	0.10	0.91
CL(18:0/18:0/18:0/18:0)	[M−2H](2H)	40	0.91	N/A	0.89
DG(18:0/20:4/0:0)	[M+Na]^+^	40	0.18	0.00	N/A
PC(16:0/16:0)	[M+H]^+^	40	0.88	0.07	0.13
TG(18:1/18:1/18:2)	[M+NH4]^+^	40	0.78	0.01	0.84

**Table 4:** Comparison of CFM-ID 3.0, CFM-ID 2.0, and MS-FINDER scoring functions upon identification of 185 compounds from 208 ESI-MS/MS spectra. Reported are the total number of challenges in which the corresponding implementation of the scoring function ranked the query compound in the top 1, top 3, and top 10. The average and median ranks for the query compound are also reported. A chemical classification is assessed as correct if the predicted category matches a category originally assigned by ClassyFire. N/A, not applicable; * performance when applied over the expanded spectral library database including the 208 experimental ESI-MS/MS from the CASMI 2016 contest (category 3).

Version	# Top 1	# Top 3	# Top 10	Average Rank	Median Rank	# Correct Classifications
*CFM-ID* 3.0	149	194	204	1.8	1	168
*CFM-ID 2.0-2019*	123	171	201	2.4	1	N/A
*CFM-ID 2.0-2016*	120	160	182	13.64	1	N/A
*MS-FINDER*	146	162	174	6.4	1	N/A
*CFM-ID* 3.0 *	208	208	208	1	1	N/A
*CFM-ID 2.0-2019* *	208	208	208	1	1	N/A

## Supplementary Materials

The following are available online at https://www.mdpi.com/2218-1989/9/4/72/s1.
