# Supplementary material for: CFM-ID 3.0: Significantly Improved ESI-MS/MS Prediction and Compound Identification
Source: Metabolites. 2019 Apr 13;9(4):72. doi: 10.3390/metabo9040072 (PMC6523630; doi:10.3390/metabo9040072)
Supplement: Supplementary file 1 [file metabolites-09-00072-s001.pdf]

metabolites

MDPI

Article

CFM-ID 3.0: Significantly Improved ESI-MS/MS Prediction Using a Hybrid In Silico Fragmentation Model with Metadata

1. Compound acquisition

All of the lipid standards were purchased from either Cayman Chemical (Ann Arbor, MI, USA) or Avanti Polar Lipids (Alabaster, AL, USA). More specifically, Glycerol Tridocosahexaenoyl, DL-Palmitoylcarnitine (chloride), 1-Palmitoyl-3-oleoyl-*sn*-glycero-2-PE, Lyso-PC, 1-Palmitoyl-2-oleoyl-*sn*-glycero-3-phosphate, Cholesteryl heptadecanoate, 2-Linoleoyl Glycerol, 1-Stearoyl-2-Arachidonoyl-*sn*-Glycerol, C-16 Ceramide, Palmitoyl Sphingomyelin, 1-Palmitoyl-2-linoleoyl PE, and 1-Octadecyl Lysophosphatidic Acid (sodium salt) were purchased from Cayman Chemical; 1-palmitoyl-2-oleoyl-*sn*-glycero-3-phospho-L-serine (sodium salt), 1',3'-bis[1,2-dioleoyl-*sn*-glycero-3-phospho]-*sn*-glycerol (sodium salt), and 1-palmitoyl-2-oleoyl-sn-glycero-3-phosphocholine were purchased from Avanti Polar Lipids, Inc.

2. Preparation of the reference solutions

15 different lipid reference solutions were prepared. The preparation of each is summarized below:

Glycerol Tridocosahexaenoyl: Glycerol Tridocosahexaenoyl was dissolved in chloroform to 5 mg/mL, and further diluted to 5 μg/mL by methanol/water (50/50) containing 0.1% formic acid.

DL-Palmitoylcarnitine (chloride): DL-Palmitoylcarnitine (chloride) was dissolved in ethanol to 5 mg/mL, and further diluted to 5 μg/mL by methanol/water (50/50) containing 0.1% formic acid.

1-Palmitoyl-3-oleoyl-sn-glycero-2-PE: 1-Palmitoyl-3-oleoyl-sn-glycero-2-PE was dissolved in chloroform to 2.5 mg/mL, and further diluted to 5 μg/mL by methanol/water (50/50) containing 0.1% formic acid.

Lyso-PC: Lyso-PC was dissolved in PBS buffer to 1 mg/mL, and further diluted to 5 μg/mL by methanol/water (50/50) containing 0.1% formic acid.

1-Palmitoyl-2-oleoyl-sn-glycero-3-phosphate: 1-Palmitoyl-2-oleoyl-sn-glycero-3-phosphate was dissolved in chloroform to 5 mg/mL, and further diluted to 5 μg/mL by methanol/water (50/50) containing 0.1% formic acid.

Cholesteryl heptadecanoate: Cholesteryl heptadecanoate was dissolved in chloroform to 5 mg/mL, and further diluted to 5 μg/mL by methanol/water (50/50) containing 0.1% formic acid.

2-Linoleoyl Glycerol: 2-Linoleoyl Glycerol was dissolved in acetonitrile to 5 mg/mL, and further diluted to 5 μg/mL by methanol/water (50/50) containing 0.1% formic acid.

1-Stearoyl-2-Arachidonoyl-sn-Glycerol: 1-Stearoyl-2-Arachidonoyl-sn-Glycerol was dissolved in methyl acetate to 10 mg/mL, and further diluted to 5 μg/mL by methanol/water (50/50) containing 0.1% formic acid.

C-16 Ceramide: C-16 Ceramide was dissolved in DMF to 0.1 mg/mL, and further diluted to 5 μg/mL by methanol/water (50/50) containing 0.1% formic acid.

Palmitoyl Sphingomyelin: Palmitoyl Sphingomyelin was dissolved in ethanol to 5 mg/mL, and further diluted to 5 μg/mL by methanol/water (50/50) containing 0.1% formic acid.

1-Palmitoyl-2-linoleoyl PE: 1-Palmitoyl-2-linoleoyl PE was dissolved in chloroform to 10 mg/mL, and further diluted to 5 μg/mL by methanol/water (50/50) containing 0.1% formic acid.

1-Octadecyl Lysophosphatidic Acid (sodium salt): 1-Octadecyl Lysophosphatidic Acid (sodium salt) was dissolved in ethanol to 5 mg/mL, and further diluted to 5 μg/mL by methanol/water (50/50) containing 0.1% formic acid.

1-palmitoyl-2-oleoyl-sn-glycero-3-phospho-L-serine (sodium salt): 1-palmitoyl-2-oleoyl-sn-glycero-3-phospho-L-serine (sodium salt) was dissolved in ethanol to 5 mg/mL, and further diluted to 5 μg/mL by methanol/water (50/50) containing 0.1% formic acid.

1',3'-bis[1,2-dioleoyl-sn-glycero-3-phospho]-sn-glycerol (sodium salt): 1',3'-bis[1,2-dioleoyl-sn-glycero-3-phospho]-sn-glycerol (sodium salt) was dissolved in ethanol to 5 mg/mL, and further diluted to 5 μg/mL by methanol/water (50/50) containing 0.1% formic acid.

1-palmitoyl-2-oleoyl-sn-glycero-3-phosphocholine: 1-palmitoyl-2-oleoyl-sn-glycero-3-phosphocholine was dissolved in chloroform to 5 mg/mL, and further diluted to 5 μg/mL by methanol/water (50/50) containing 0.1% formic acid.

3. Instrumentation and Parameterization

An AB Sciex QTrap 4000 mass spectrometer (Framingham, MA, USA) was used to collect all the MS/MS spectra. The samples were introduced via direct infusion with Harvard syringe pump at flow rate of 10 μL/min. Positive & negative instrument mode parameters were set as follows:

Scan Type: Enhanced Product Ion (EPI)
Polarity: Positive
Scan Mode: Profile
Ion Source: Turbo Spray
Resolution of Q1: Unit
Scan Rate: 1000 amu/s
MR Pause: 5.0070 msec
Q0 trapping: No
MCA: Yes
LIT fill time: 20 msec
Dynamic Fill Time: On
CUR: 10
CAD: High
IS: 4500
GS1: 20
GS2: 0
ihe: On

DP: 140
CES: 0
CE: 10/20/30/40 (some lipids were scanned up to 60)

Scan Type: Enhanced Product Ion (EPI)
Polarity: Negative
Scan Mode: Profile
Ion Source: Turbo Spray
Resolution of Q1: Unit
Scan Rate: 1000 amu/s
MR Pause: 5.0070 msec
Q0 trapping: No
MCA: Yes
LIT fill time: 20 msec
Dynamic Fill Time: On
CUR: 10
CAD: High
IS: -4500
GS1: 20
GS2: 0
ihe: On
DP: -110
CES: 0
CE: -10/-20/-30/-40

4. Measurements and Data Extraction

Lipid standard solutions were introduced into the QTrap 4000 mass spectrometer via direct infusion with a Harvard syringe pump at a flow rate of 10 μL/min. For each lipid standard solution, an enhanced MS (EMS) scan was first conducted to identify precursor ions with high abundance (e.g., M+H, M+Na, M+NH4, M-H, etc.). Enhanced product ion (EPI) scans for each precursor ion were then conducted to generate the MS/MS spectra with different collision energy (CE) levels. MS/MS spectra for most of the lipids were collected for CE +/- 10 to 40 eV, while spectra collected for some lipids were shifted up to CE levels of +/- 60 eV depending on the observed fragmentation patterns. For example, if the precursor ion signal in the MS/MS spectrum was still high at a CE level of 40 eV, the next CE level above would then be tested until a CE level was found in which the precursor ion signal was very low or almost zero.

MS/MS spectra for each lipid standard were collected for both positive and negative ion modes, with different collision energy (CE) levels, i.e. +10/+20/+30/+40, -10/-20/-30/-40, eV etc. Each EPI scan was conducted and monitored until a stable signal was observed, then the "Acquire" function in the Sciex Analyst software was applied to collect each MS/MS spectrum for one minute. Molecular masses were first picked via the EMS scan if they showed sufficiently high abundance. For those not seen in the EMS scan, calculations based on the molar mass of the native lipid being analyzed were conducted.

© 2019 by the authors. Submitted for possible open access publication under the terms and conditions of the Creative Commons Attribution (CC BY) license (http://creativecommons.org/licenses/by/4.0/).
